# Supplementary material for: Haplotypes of the tRNAleu-COII mtDNA Region in Russian Apis mellifera Populations
Source: Animals (Basel). 2023 Jul 24;13(14):2394. doi: 10.3390/ani13142394 (PMC10376158; doi:10.3390/ani13142394)
Supplement: Supplementary file 1 [file animals-13-02394-s001.zip › Supplementary table S1.docx]

**Supplementary table S1 *tRNAleu-COII* haplotypes in Russian *Apis mellifera* L. samples**

| **Region** | ***tRNAleu-COII* allelic variants** | ***tRNAleu-COII* haplotypes** |
| --- | --- | --- |
| Altai Krai | 1 Q, 9 PQQ | 1 С2с, 9 M17j |
| Kirov Oblast | 4 Q, 6 PQQ | 3 C2c, 1 C2i2, 4 M17j, 2M17j |
| Krasnodar Krai | 13 Q | 10 C2j, 1 C2jf, 1 C2jd, 1 C2je |
| Leningrad Oblast | 1 Q, 3 PQQ, 8 PQQQ | 1 C2c, 3 M17j, 3 M4h', 5 M4g' |
| Nizhny Novgorod Oblast | 4 Q, 1 PQQ | 4 C2c, 1 M17j |
| Novgorod Oblast | 6 Q, 1 PQQ, 3 PQQQ | 3 C2c, 1 C2l, 1 C1, 1 C4a, 1 M17j, 3 M4h' |
| Omsk Oblast | 3 Q, 7 PQQ | 2 C2c, 1 C2j, 7 M17j |
| Orenburg Oblast | 2 Q | 2 C2c |
| Perm Krai | 1 Q, 46 PQQ | 1 C2ja, 2 M17q, 6 M17k, 38 M17j |
| Primorsky Krai | 2 Q | 2 C2 |
| Pskov Oblast | 4 PQQ, 2 PQQQ | 4 M17j, 2 M4g' |
| Republic of Adygea | 12 Q | 3 C2c, 1 C2j, 1 C2, 6 C1, 1 C1j |
| Republic of Bashkortostan | 1Q, 43 PQQ, 17 PQQQ | 1 C3, 36 M17j, 1 M17j2, 1 M17n, 1 M17k, 1 M17o, 1 M17r, 2 M17l, 15 M4g’, 1 M4i’, 1 M4j’ |
| Republic of Tatarstan | 10 PQQ | 10 M17j |
| Udmurt Republic | 10 PQQ | 7 M17j, 2 M17k, 1 M17p |
| Ryazan Oblast | 16 Q | 3 C2, 8 C2c, 2 C3, 3 C2ja |
| Saratov Oblast | 7 PQQ | 7 M17j |
| Sverdlovsk Oblast | 2 Q, 4 PQQ, 1 PQQQ | 2C2, 3 M17j, 1 M17j, 1 M4g’ |
| Tver Oblast | 3 Q, 16 PQQ | 1 C2, 1 C2c, 1 C2i2, 15 M17j, 1 M17s |
| **Total:** | **269** | **71 C lineage, 198 M lineage** |
