# Supplementary figures and images for: Haplotypes of the tRNAleu-COII mtDNA Region in Russian Apis mellifera Populations
Source: Animals (Basel). 2023 Jul 24;13(14):2394. doi: 10.3390/ani13142394 (PMC10376158; doi:10.3390/ani13142394)

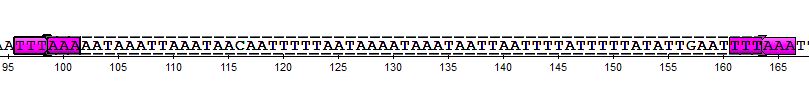

Supplement: Supplementary file 1 [file animals-13-02394-s001.zip › Figure S1.JPG]

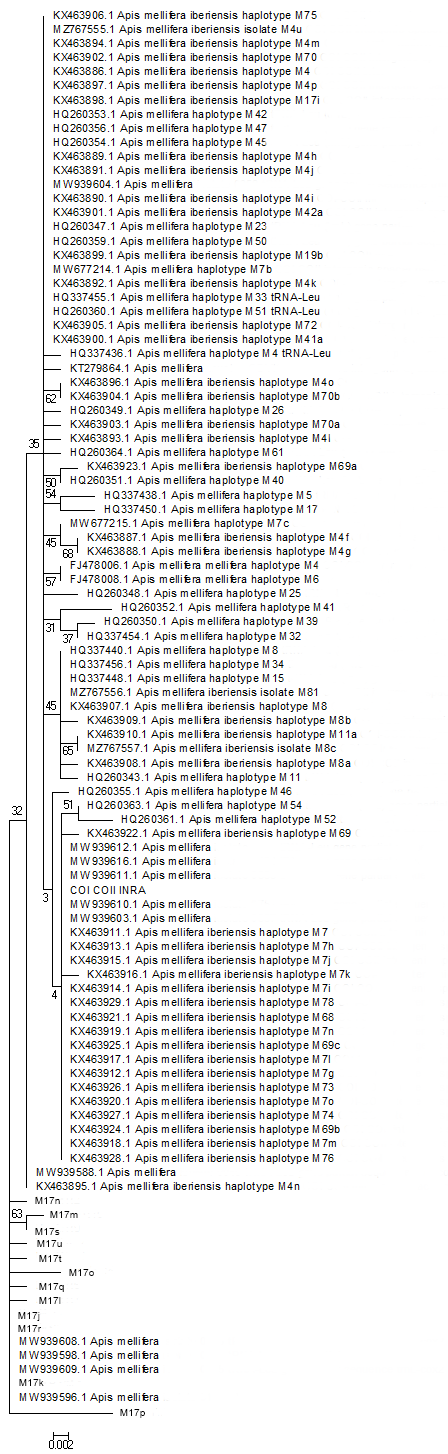

Supplement: Supplementary file 1 [file animals-13-02394-s001.zip › Figure S2.png]

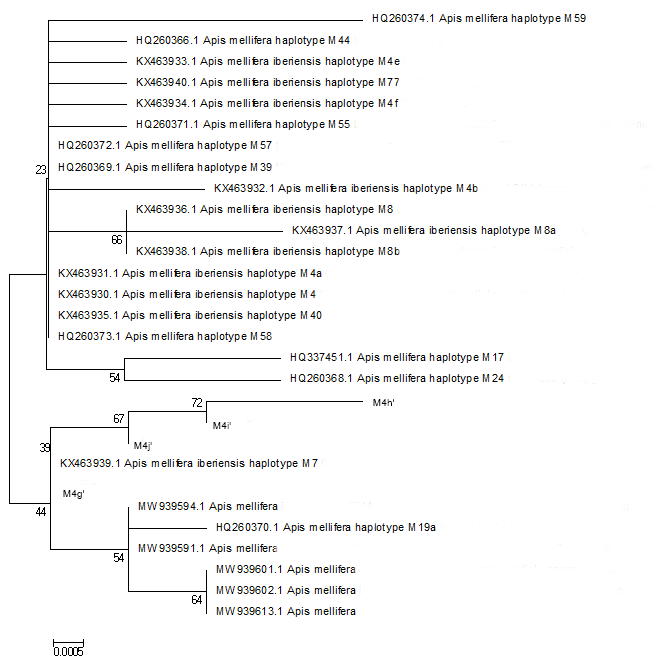

Supplement: Supplementary file 1 [file animals-13-02394-s001.zip › Figure S3.png]
